# Assessment of Functional Outcome Predictors in Patients Undergoing Lumbar Interbody Fusion Surgery: A Single-Centre Analysis

**DOI:** 10.7759/cureus.23529

**Published:** 2022-03-27

**Authors:** Renata Marques, Sara Gomes, João Nogueira, Miguel Afonso, Nubélio Duarte

**Affiliations:** 1 Neurosurgery, Hospital de Braga, Braga, PRT; 2 Medicine, Life and Health Sciences Research Institute (ICVS), Braga, PRT

**Keywords:** radicular pain, oswestry disability index, lumbar stenosis, lumbar spondylolisthesis, lumbar pain, lumbar interbody fusion

## Abstract

Introduction

Lumbar interbody fusion is a surgical modality performed in selected patients with low back and radicular pain not responding to medical therapy. We aim to evaluate the main predictors of functional outcome, assessed through Oswestry Disability Index (ODI), in patients submitted to a lumbar interbody fusion.

Methods

A sample of 33 patients undergoing lumbar interbody fusion at a neurosurgery department between 2017 and 2020 was selected. In order to assess functional status, ODI was applied before and after surgery. Data related to patients' medical history, current disease, and surgery performed were collected from the clinical process.

Results

In our cohort, functional improvement (pre-surgery ODI - post-surgery ODI) averaged 34.4 ± 23 points, suggesting robust surgical efficacy. We find patients with severe disability or worse to display relevant amelioration of their functional scores (p<0.0001), suggesting that these can benefit from lumbar interbody fusion surgery. The female gender (p=0.007) predicts a better outcome, which was surprising as no sex differences in lumbar fusion outcomes have been reported. Conversely, early symptom recurrence (p=0.015) and need for revision surgery (p=0.032) were found to be negative predictors of post-surgical functional outcome. Rapid return to the activities of daily living (p=0.001) and to work (p=0.002) was associated with post-surgical functional improvement. The underlying diagnosis that led to surgical referral and surgical modality did not affect the functional outcome in our patient cohort. Importantly, patients with previous lumbar surgeries had similar improvements to those who had never been operated on.

Conclusions

Severely disabled patients submitted to lumbar interbody fusion showed significant functional improvement, regardless of the referral diagnosis or the existence of previous lumbar surgeries. Additionally, sustained functional improvement resulted in a return to an active life.

## Introduction

Chronic low back pain and/or radiculopathy are prevalent symptoms in the clinical setting. These can be refractory to medical therapy in pathologies such as lumbar stenosis, with or without spondylolisthesis, and post-surgical instability syndromes. Lumbar interbody fusion is a surgical modality used to treat these conditions [1,2].

Lumbar canal stenosis arises from posterior vertebral osteophytes formation, ligamentum flavum, and facet joints hypertrophy. It can be central or foraminal, and some patients might have associated spondylolisthesis [3]. Spondylolisthesis can be isthmic or degenerative. The former mainly affects young adults and structural alterations usually occur in the L5-S1 segment. The latter is the most frequent etiology of spondylolisthesis, and females over the age of 50 are the most affected, involving generally the L4-L5 segment. Surgical management may involve decompression associated with fusion through a posterior approach, particularly in the presence of significant spinal or foraminal stenosis with clinical or radiological pre-surgery instability [4,5].

Post-surgical instability syndromes are characterized by the presence of low back pain in patients previously subjected to laminectomy or discectomy. Interbody fusion procedures are indicated in refractory cases to medical therapy [6,7], to stabilize the painful segment, achieve neural decompression, and correct the deformity.

There are several surgical approaches to fuse the lumbar segments, such as anterior, posterior, lateral, oblique, or transforaminal approaches. The current study focuses on patients submitted to classic posterior lumbar interbody fusion (PLIF) and transforaminal lumbar interbody fusion (TLIF) [1]. PLIF has several advantages over the other techniques, as it allows good visualization of the nerve roots without compromising blood flow, re-establishes interbody height, and warrants adequate decompression of neural structures while maintaining appropriate support, allowing a fusion of 360º with a single incision. Yet, excessive nerve or dural sac retraction and disruption of muscle integrity can cause pain, numbness, and/or neurological deficits after the procedure. TLIF might injure soft tissues due to neuromuscular retraction as well, but it has the advantage of providing easy access to the posterior structures, directly and unilaterally, decreasing the surgical trauma of the nerve roots [1,8].

Despite the widespread use of interbody lumbar fusion surgeries, the indications for intervention are at times nuanced. Therefore, we performed an Oswestry Disability Index (ODI)-based questionnaire to evaluate surgical impact in our cohort, to understand the functional outcome predictors, and the ability to return to work and Activities of Daily Living (ADL).

## Materials and methods

Study design and population

A retrospective and observational study was carried out to evaluate the initial experience of a single-centre neurosurgical department. The study cohort consists of 35 patients submitted to classic PLIF or TLIF in a neurosurgery department of a district hospital between January 2017 and August 2020.

Patients diagnosed with lumbar spinal stenosis, with or without spondylolisthesis, and post-surgical instability syndromes undergoing classic PLIF or TLIF willing to participate in the study were included.

Patients with a diagnosis different from the above-mentioned or patients who did not accept to participate in the study were excluded.

Data collection and description

An informed consent was provided to patients. Then, data were collected from the clinical records and by telephone interview. Phone calls were used to collect data regarding the functional assessment of patients, through the application of a questionnaire based on the Oswestry Disability Index.

Data collected from the clinical records comprised are age and gender of patients, their comorbidities, whether they were previously submitted to lumbar surgery, current disease diagnosis, and associated symptoms, such as low back pain, radicular symptoms, and/or neurological deficits pre-surgery. According to their comorbidities, patients were classified based on the American Society of Anesthesiologists (ASA) physical status classification. Information about surgery details and its complications, the duration of the post-surgery hospitalization, symptomatic relapse, and the need for revision surgery was collected as well.

The clinical outcome of lumbar fusion interventions can be assessed using the Oswestry Disability Index (ODI) version 2.0, which evaluates patient’s symptomatology, functionality, and quality of life. It is a reliable, valid, and simple tool [9]. It has ten sections: pain intensity, personal care, lifting, walking, sitting, standing, sleeping, sex life, social life and travelling. Each of which with six options scored from zero to five. The sum of the scores from all sections multiplied by two results in the percentage of disability. Patients fit into one of five categories: minimal disability (0% to 20%), moderate disability (21% to 40%), severe disability (41% to 60%), crippled (61% to 80%) and bedridden patients (81% to 100%).

ODI assessment was performed after the intervention, addressing retrospectively the functional disability before and after the lumbar interbody fusion surgery. Information about the return to ADL and to work was also obtained with this questionnaire. We defined functional improvement as the percentual difference between pre-surgery ODI and post-surgical ODI.

Statistical analysis

Data were analyzed using the SPSS (IBM Corp. Released 2020. IBM SPSS Statistics for Windows, Version 27.0. Armonk, NY: IBM Corp). Mean (M) and standard deviation (SD) or frequencies were used for descriptive statistics. Independent-samples t-test and one-way analysis of variance (ANOVA) were used to compare functional improvement between groups. Pearson's Correlation was performed to correlate functional improvement with other continuous variables. For all models, statistical significance was defined as p-value less than 0.05, with a confidence interval of 95%.

## Results

**Figure 1:**
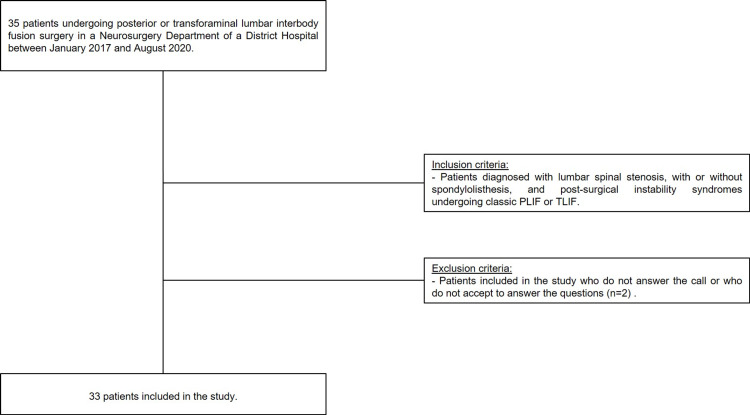
Flow Chart Diagram representing the Study Design.

A sample of 33 patients submitted to classic PLIF and TLIF was selected, after the inclusion and exclusion criteria were applied. The flow chart diagram representing the study design is shown in Figure 1.

Descriptive analysis of the population

Table 1 summarizes the descriptive analysis of the patient’s characteristics included in the study. Of the 33 patients involved in this project, 21 were female (63.6%) with an age range between 38 and 80 years (54.4±9.3). At the time of surgery, most of the patients were non-smokers (31; 93.9%) and only a few were obese (4; 12.1%). Most participants were ASA 2 (18; 54.5%) in terms of comorbidity. Fourteen patients had between one to three previous lumbar surgeries, in more detail six, five, and three patients underwent one, two, and three previous lumbar surgeries, respectively.

**Table 1:** Preoperative, intraoperative and postoperative details. ADL: Activities of Daily Living; ASA: American Society of Anesthesiologists physical status classification; PLIF: Posterior Lumbar Interbody Fusion; TLIF: Transforaminal Lumbar Interbody Fusion

Details			Values
Total no. of patients			33
Pre-surgery details	Age		54.36 ± 9.266
	Sex	Female	21 (63.6%)
		Male	12 (36.4%)
	Smoking		2 (6.1%)
	Obesity (BMI≥30)		4 (12.1)
	ASA	1	11 (33.3%)
		2	18 (54.5%)
		3	4 (12.1%)
	Previous lumbar surgery		14 (42.4%)
	Diagnosis	Lumbar stenosis with Spondylolisthesis	21 (63.6%)
		Lumbar stenosis without Spondylolisthesis	5 (15.1%)
		Post-surgical Instability	7 (21.2%)
	Spondylolisthesis’ Degree	Grade 1	11 (33.3%)
		Grade 2	10 (30.3%)
	Radicular Symptoms		32 (97.0%)
	Low Back Pain		31 (93.9%)
	Neurological Deficits		9 (27.3%)
Surgery details	Type of Surgery	TLIF	19 (57.6%)
		PLIF	14 (42.4%)
	Segments	L3-L4	1 (3.0%)
		L4-L5	14 (42.4%)
		L4-L5 and L5-S1	3 (9.1%)
		L5-S1	15 (45.5%)
	Interbody Fusion Material	Cage	32 (97.0%)
		Bone	1 (3.0 %)
	Surgical Complications	Durotomy	2 (6.1%)
Post-surgery details	Bleeding		1 (3.0%)
	Medical Complications		6 (18.2%)
	Symptomatic Relapse		9 (27.3%)
	Need for revision Surgery		2 (6.1%)
	Post-Surgery Hospitalization Time (days)		4.849 ± 2.884

In our cohort, lumbar spinal stenosis with spondylolisthesis (63.6%) was the major diagnosis followed by post-surgical instability (21.2%). Lumbar spinal stenosis without spondylolisthesis was the least prevalent (15.1%). Grade I spondylolisthesis was most frequently found (33.3%).

Radicular symptoms were present in all but one patient (32; 97.0%). Low back pain was the commonest manifestation (31; 93.9%) and nine patients had motor or sensory neurological deficits (27.3%) at presentation.

Most patients were submitted to TLIF (19; 57.6%) and the remaining 14 underwent PLIF (42.4%). L4-L5 and L5-S1 were the segments most often intervened. To preserve disc height, a cage was used in almost all patients (97.0%), except in one where a bone graft was preferred.

Post-surgery hospitalization lasted from two to fourteen days (4.9±2.9). Medical complications, such as urinary tract infections, fever, and allergic reactions occurred in six patients. Surgical complications such as durotomy occurred in two patients and bleeding in one.

Nine patients submitted to lumbar interbody fusion had a symptomatic relapse after the intervention, although these symptoms were less intense when compared to those before intervention. Revision surgery was required in two patients.

Determining the functional improvement upon lumbar interbody fusion

**Figure 2:**
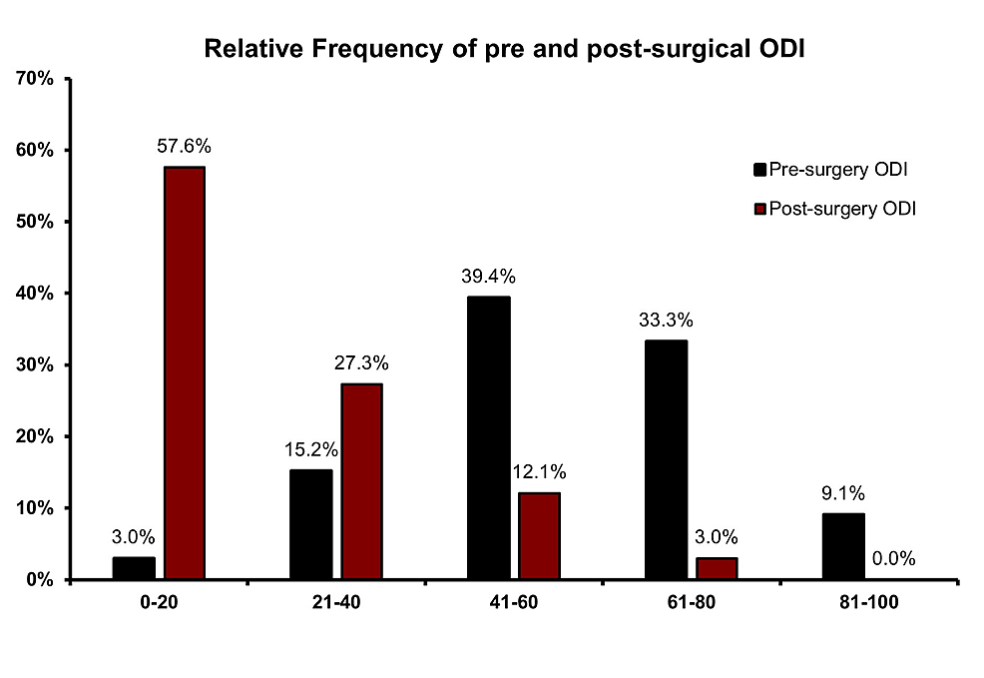
Bar Chart illustrating the Relative Frequency (%) of Pre and Post-surgery Oswestry Disability Index (ODI)'s score in patients submitted to lumbar interbody fusion.

To address whether lumbar interbody fusion surgery led to functional improvement in our population, we determined the ODI-score before and after surgery. As shown in Figure 2, 27 patients (81.8%) had severe disability or worse ascertained by the pre-surgical ODI-score. Reversely, post-surgical ODI analysis showed that 28 patients (84.9%) had minimal or moderate disability. To better quantify this functional improvement, we calculated the difference between pre-surgery ODI and post-surgery ODI for each patient (Table 2). Upon lumbar interbody fusion surgery, there was a significant decrease, on average of 34.4±23 points, in the ODI score. The values obtained ranged from -36% to 84%. Moreover, 30 patients were able to return to pre-symptomatic ADL (90.9%, p<0.001) and 17 patients out of 23 previously active labors returned to work (73.9%, p<0.001) (Table 3). Both were significantly associated with ODI-reported functional improvement. Patients able to return to ADL and to work had a mean improvement of 38.3 ± 18.7 and 41.4 ± 17.5, respectively (Table 3). Overall, these results suggest a high surgical efficacy in improving patient symptoms and quality of life.

**Table 2:** Surgical functional improvement.

Functional Improvement	Mean ± SD	p-value
Pre-surgery ODI – Post-surgery ODI	34.364 ± 22.986	p<0.001

**Table 3:** Analysis of Variables associated with Functional Improvement.

Variables		n (%)	Functional Improvement (Mean±SD)
Return to ADL (p<0.001)	No	3 (9.1%)	-5.333 ± 28.378
	Yes	30 (90.9%)	38.333 ± 18.659
Return to Work (p<0.001)	No	6 (26.1%)	5.000 ± 22.441
	Yes	17 (73.9%)	41.412 ± 17.46

Predictors of functional improvement in our cohort

To determine the predictors of functional improvement in our cohort we analyzed patient’s characteristics and their surgical outcome defined by Pre-surgery ODI minus Post-surgery ODI (Table 4). We have found that sex (p=0.007), pre-surgery ODI (p<0.001), symptomatic relapse (p=0.015) and need for revision surgery (p=0.032) are predictors of functional improvement (Table 4, 5).

**Table 4:** Analysis between Patients’ variables and Functional Improvement. ADL: Activities of Daily Living; ASA: American Society of Anesthesiologists physical status classification; ODI: Oswestry Disability Index.

Variables	p values
Sex	p=0.007
Age	p=0.280
Smoking	p=0.337
Obesity	p=0.742
ASA	p=0.436
Previous Lumbar Surgery	p=0.442
Diagnosis	p=0.266
Isthmic vs Degenerative Spondylolisthesis	p=0.243
Degree of Spondylolisthesis	p=0.873
Neurological Deficits	p=0.580
PLIF vs TLIF	p=0.177
Segments	p=0.556
Surgical Complications	p=0.439
Medical Complications	p=0.377
Post-Surgery Hospitalization Time	p=0.124
Symptomatic Relapse	p=0.015
Revision Surgery	p=0.032
Pre-surgery ODI	p<0.001

**Table 5:** Analysis of Predictors of Functional Improvement. ADL: Activities of Daily Living; ODI: Oswestry Disability Index

Variables		Functional Improvement (Mean±SD)
Sex	Female	42.191 ± 18.920
	Male	20.667± 23.761
Symptomatic Relapse	No	40.167 ± 20.069
	Yes	18.889 ± 24.189
Revision Surgery	No	36.516 ± 22.012
	Yes	1.000 ± 1.414
Pre-surgery ODI	0-20	-36.000
	21-40	21.200 ± 6.723
	41-60	33.385 ± 13.150
	61-80	38.909 ± 20.087
	81-100	67.333 ± 25.482

In our study population, women have a greater ODI improvement (42.2 ± 18.9) when compared to men (Table 5). Patients without symptomatic relapse (40.2 ± 20.1) or need for revision surgery (36.5 ± 22.0) display robust functional improvement after intervention.

Importantly, patients with higher pre-surgery ODI (61% to 80% and 81% to 100%), which means a greater dysfunctionality before surgery, have a substantial functional improvement (38.9 ± 20.1 and 67.3 ± 25.5, respectively) (Table 5).

On the other hand, age (p=0.28), obesity (p=0.742), previous lumbar surgery (p=0.973) and surgery referral diagnosis (p=0.266) did not impact functional improvement (Table 4).

## Discussion

The current study aims to understand the predictors of functional improvement, using the ODI questionnaire, in patients submitted to interbody lumbar fusion. We find that most patients had a substantial functional improvement with the intervention, with a mean ODI decrease of 34.4 points. This supports the idea that this surgical intervention has a positive impact on the patient’s quality of life. Moreover, 57.6% of patients have a post-surgery ODI of 0% to 20%. Our results are consistent with previously published data on quality of life improvement after lumbar fusion surgery [2,10].

Functional improvement can be affected by variables related to the patient, their medical history, current diagnosis, and surgical intervention per se. Therefore, we wanted to analyze the impact of several variables in functional improvement. We found four of those affecting the functional outcome significantly.

Regarding variables related to patient characteristics, only sex was a significant predictor of ODI variation. Our results showed that women report a stronger functional improvement measured by ODI. Past studies about minimal invasive lumbar fusion outcomes reported that gender does not significantly affect post-surgery ODI [11,12]. The need for revision surgery in two men could justify the lesser improvement of males in our study, but it would be interesting to understand other factors associated with a biological sex that may explain this result.

Factors such as age, body mass index, and medical comorbidities do not affect the functional outcome in our study. These conclusions are in accordance with data reported by other authors [13-15]. In our population, there were only two smokers, which limits this specific analysis. Also, neurological deficits at presentation were not useful at predicting the patients that would benefit the most from surgery, it can however affect recovery [16].

Interestingly, 14 patients (42%) had previous lumbar surgeries, and their improvement was comparable to lumbar surgery-naive patients. To our knowledge, we are the first to report a comparable outcome after interbody lumbar fusion surgery between naive vs. previously intervened patients [17,18]. Therefore, our results suggest that patients previously submitted to lumbar surgery are also good candidates for lumbar fusion procedures.

The referral diagnosis for surgery, namely lumbar spinal stenosis with or without spondylolisthesis and post-surgical instability, did not affect the post-surgical functional outcome, as patients in each group did not show significant differences in our ODI assessment. This is in accordance with other authors [19].

Most variables related to the surgery, such as type of interbody fusion performed, segments intervened, and duration of post-surgery hospitalization, do not influence the functional outcome in our cohort, as also reported by others [13,20]. Srikanth et al. and Mobbs et al. [1,20] both report that the type of surgery does not impact the outcome, so the surgical approach is done according to advantages, disadvantages, and limitations at the surgical level tailored for each patient. In our cohort, patients undergoing TLIF and PLIF had similar results supporting the previously published claims. Although our study does not show an impact of surgical complications in the ODI quantification, Abduljabbar et al. show a correlation between blood loss and worse functional outcomes [13]. We did not observe this, probably due to the limited number of surgical complications.

Patients with symptomatic relapse and need for revision surgery exhibit, as expected, a minor improvement in functional status. Therefore, these two aspects, are natural significant predictors of poor functional improvement in the post-operative period.

Noteworthy, our results suggest that a worse dysfunction before surgery predicts a greater improvement after the intervention, as patients that were significantly impaired at the time of referral, benefited the most from surgery. Although one study reported that pre-surgery ODI does not correlate with functional outcome [13], patients with higher ODI scores appear to improve more with interbody lumbar fusion in another work [21]. Therefore, patients with severe deterioration of functional status before intervention appear to be good candidates for interbody lumbar fusion.

The small sample size (n=33) limits the broader generalization of our conclusions. Yet, we believe that this study reinforces the applicability of classic PLIF or TLIF to treat patients with lumbar spinal stenosis, with or without spondylolisthesis, and post-surgical instability syndromes. Importantly, patients with the worse functional conditions before the intervention, regardless of having previous lumbar surgery, show major improvements with interbody lumbar fusion procedures.

## Conclusions

Our study demonstrates that classic PLIF and TLIF have good functional outcomes in patients with lumbar spinal stenosis, with or without spondylolisthesis, and post-surgical instability syndromes. This reinforces the indication to perform these procedures in selected patients, as they seem to add quality of life and resolve burdensome symptoms.

We were able to find relevant ODI predictors between the variables under examination. The female sex, higher pre-surgical ODI were positive predictors of functional improvement. On the other hand, we find that symptomatic recurrence and need for revision surgery were negative predictors.

## References

[1] Mobbs RJ, Phan K, Malham G, Seex K, Rao PJ (2015). Lumbar interbody fusion: techniques, indications and comparison of interbody fusion options including PLIF, TLIF, MI-TLIF, OLIF/ATP, LLIF and ALIF. J Spine Surg.

[2] Luckenbill D, Goswami R, Grannis KA, O'Neill J, Goswami T (2015). Retrospective lumbar fusion outcomes measured by ODI sub-functions of 100 consecutive procedures. Arch Orthop Trauma Surg.

[3] Binder DK, Schmidt MH, Weinstein PR (2002). Lumbar spinal stenosis. Semin Neurol.

[4] Bhalla A, Bono CM (2019). Isthmic lumbar spondylolisthesis. Neurosurg Clin N Am.

[5] Bydon M, Alvi MA, Goyal A (2019). Degenerative lumbar spondylolisthesis: definition, natural history, conservative management, and surgical treatment. Neurosurg Clin N Am.

[6] Teixeira MJ, Yeng LT, Garcia OG, Fonoff ET, Paiva WS, Araujo JO (2011). Failed back surgery pain syndrome: therapeutic approach descriptive study in 56 patients (Article in Portuguese). Rev Assoc Med Bras (1992).

[7] Erdem MN, Erken HY, Aydogan M (2018). The effectiveness of non-surgical treatments, re-discectomy and minimally invasive transforaminal lumbar interbody fusion in post-discectomy pain syndrome. J Spine Surg.

[8] Martinelli TC, Effgen EA, Brazolino MA, Cardoso IM, Maia TC, Jacob Junior C (2018). Evaluation of the discal height gain and lumbar lordosis variation obtained by the techniques of transforaminal and posterior lumbar intersomatic fusion. Rev Bras Ortop.

[9] Yates M, Shastri-Hurst N (2017). The oswestry disability index. Occup Med.

[10] Rousseau MA, Lazennec JY, Bass EC, Saillant G (2005). Predictors of outcomes after posterior decompression and fusion in degenerative spondylolisthesis. Eur Spine J.

[11] Hoffmann CH, Kandziora F (2020). Minimally invasive transforaminal lumbar interbody fusion (Article in German). Oper Orthop Traumatol.

[12] Khechen B, Haws BE, Patel DV, Cardinal KL, Guntin JA, Singh K (2019). Does gender influence postoperative outcomes in minimally invasive transforaminal lumbar interbody fusion?. Clin Spine Surg.

[13] Abduljabbar FH, Makhdom AM, Rajeh M (2017). Factors associated with clinical outcomes after lumbar interbody fusion with a porous nitinol implant. Global Spine J.

[14] Gatot C, Liow MH, Goh GS (2022). Smoking is associated with lower satisfaction in nondiabetic patients undergoing minimally invasive single-level transforaminal lumbar interbody fusion. Clin Spine Surg.

[15] Owens RK 2nd, Djurasovic M, Onyekwelu I, Bratcher KR, McGraw KE, Carreon LY (2016). Outcomes and revision rates in normal, overweight, and obese patients 5 years after lumbar fusion. Spine J.

[16] Cook CE, Frempong-Boadu AK, Radcliff K, Karikari I, Isaacs R (2015). Older age and leg pain are good predictors of pain and disability outcomes in 2710 patients who receive lumbar fusion. HSS J.

[17] Hentenaar B, Spoor AB, de Waal Malefijt J, Diekerhof CH, den Oudsten BL (2016). Clinical and radiological outcome of minimally invasive posterior lumbar interbody fusion in primary versus revision surgery. J Orthop Surg Res.

[18] Montenegro TS, Gonzalez GA, Saiegh FA (2021). Clinical outcomes in revision lumbar spine fusions: an observational cohort study. J Neurosurg Spine.

[19] Cobo Soriano J, Sendino Revuelta M, Fabregate Fuente M, Cimarra Díaz I, Martínez Ureña P, Deglané Meneses R (2010). Predictors of outcome after decompressive lumbar surgery and instrumented posterolateral fusion. Eur Spine J.

[20] Divi SN, Schroeder GD, Goyal DK (2019). Fusion technique does not affect short-term patient-reported outcomes for lumbar degenerative disease. Spine J.

[21] Carreon LY, Glassman SD, Djurasovic M, Dimar JR, Johnson JR, Puno RM, Campbell MJ (2009). Are preoperative health-related quality of life scores predictive of clinical outcomes after lumbar fusion?. Spine (Phila Pa 1976).

